# Role of anterior segment optical coherence tomography angiography in the assessment of acute chemical ocular injury: a pilot animal model study

**DOI:** 10.1038/s41598-021-96086-0

**Published:** 2021-08-17

**Authors:** Kai Yuan Tey, Jinyuan Gan, Valencia Foo, Bingyao Tan, Meng Yuan Ke, Leopold Schmetterer, Jodhbir S. Mehta, Marcus Ang

**Affiliations:** 1 Singapore Eye Research Institute, Singapore, Singapore; 2 Duke-NUS Medical School, Singapore, Singapore; 3 Singapore National Eye Centre, 20 College Rd, Singapore, 169856 Singapore; 4 SERI-NTU Advanced Ocular Engineering (STANCE) Program, Nanyang Technological University, Singapore, Singapore; 5 School of Chemical and Biomedical Engineering, Nanyang Technological University, Singapore, Singapore; 6 Department of Clinical Pharmacology, Medical University of Vienna, Vienna, Austria; 7 Center for Medical Physics and Biomedical Engineering, Medical University of Vienna, Vienna, Austria; 8 Institute of Molecular and Clinical Ophthalmology, Basel, Switzerland

**Keywords:** Medical imaging, Prognostic markers

## Abstract

To examine the use of anterior segment-optical coherence tomography angiography (AS-OCTA) in the assessment of limbal ischemia in an animal model chemical ocular injury. We conducted a prospective study using an established chemical ocular injury model in 6 rabbits (12 eyes), dividing the cornea limbus into 4 quadrants. Chemical injury grade was induced based on extent of limbal injury (0 to 360 degrees) and all eyes underwent serial slit-lamp with AS-OCTA imaging up to one month. Main outcome measure was changes in AS-OCTA vessel density (VD) comparing injured and control cornea limbal quadrants within 24 h and at one month. AS-OCTA was able to detect differences in limbal VD reduction comparing injured (3.3 ± 2.4%) and control quadrants (7.6 ± 2.3%; *p* < 0.001) within 24 h of ocular chemical injury. We also observed that AS-OCTA VD reduction was highly correlated with the number of quadrants injured (r = − 0.89; *p* < 0.001; 95% CI − 5.65 to − 1.87). Corneal vascularization was detected by AS-OCTA in injured compared to control quadrants (10.1 ± 4.3% vs 7.0 ± 1.2%; *p* = 0.025) at 1 month. Our animal pilot study suggests that AS-OCTA was able to detect limbal vessel disruption from various severities of acute chemical insult, and in the future, could potentially serve as an adjunct in providing objective grading of acute ocular chemical injury once validated in a clinical trial.

## Introduction

Chemical injuries are one of the most common ocular emergencies accounting for 11.5–22% of ocular traumas^1^, which can cause permanent visual impairment due to secondary corneal scarring and limbal stem cell deficiency (LSCD)^2^. The extent of tissue damage to the conjunctiva, cornea and limbus are used as prognostic indicators following chemical injury^3^. Hence, accurate clinical grading of the acute severity of the injury is essential as it guides acute and long term management of such eyes. Existing clinical grading systems include the extent of limbal ischemia (disruption and loss of limbal vascular arcade architecture), which may be associated with limbal stem cell loss and subsequent corneal scarring^3–5^. Clinical assessment of limbal ischemia on slit-lamp examination is however highly subjective^6–9^.

Optical coherence tomography angiography (OCTA) is a rapid, non-contact imaging technique that can help delineate ocular vasculature^10–14^. Whilst existing OCTA systems were not specifically designed for imaging the anterior segment, they can be adapted to assess cornea or iris vasculature, i.e. anterior segment OCTA (AS-OCTA)^10,15^. This is particularly useful for imaging the vasculature of the cornea objectively^16^, in the assessment of cornea graft vascularisation^17^, incipient risks of graft rejection, severity of LSCD^18^, or glaucoma drainage bleb vascularity^19^. Previous studies have also demonstrated that OCTA images are comparable to indocyanine green angiography (ICGA) in capturing corneal vascularization using various animal models^20,21^.

Thus, AS-OCTA may be a useful objective imaging method to identify limbal involvement from acute chemical injuries, and has been used in a pilot clinical study to suggest that it may improve prognostication and guide clinical management^22^. However, as this was a clinical study it could not specifically examine the correlation between the exact extent of limbal chemical injury with AS-OCTA findings, and instead used slit-lamp examination with fluorescein staining as a surrogate. Therefore, we conducted an animal study using an established model of acute chemical injury to study the effect of chemical injury burns directly on the limbus using AS-OCTA imaging and examine changes in limbal vessel density over one month.

## Methods

This study included 12 eyes of 6 male New Zealand white rabbits, 2.0–2.5 kg, 3–4 months of age. All eyes were categorized into four quadrants (superior nasal, superior temporal, inferior nasal, inferior temporal). These quadrants were then further stratified into either the non-injury (ie. controls) or injury group, where the cornea limbus was exposed to a chemical injury model as previously described^23^. All animals were treated as per guidelines of the Association for Research in Vision and Ophthalmology’s statement for the Use of Animals in Ophthalmic and Vision Research. This study was also carried out in compliance with the ARRIVE guidelines^24^. Experimental protocols were carried out as approved by the SingHealth Institutional Animal Care and Use Committee (IACUC reference number: 202003-00130) and housed under standard laboratory conditions at the SingHealth Experimental Medical Centre, Singapore General Hospital.

### Chemical Injury technique

All procedures were performed using a previously described technique^23^, under anesthesia with an intramuscular injection of xylazine HCl (5 mg/kg) and ketamine HCl (50 mg/kg), further supplemented by topical anesthesia (0.4% oxybuprocaine HCl). A 1 N sodium hydroxide (NaOH) soaked disc-shaped 4-mm filter paper (soaked for 10 s in 50ul of NaOH) was placed on the cornea limbus for 30 s in various quadrants, as to induce various grades of chemical injury based on limbal involvement. The conjunctival sac was then rinsed with saline until a pH of 7 to 7.5 was achieved. for 4 weeks to all eyes. Topical Oxybyprocaine 0.4% and oral analgesia were administered if the rabbits were in distress, and topical antibiotics were applied (Tobramycin ointment 0.3% four times daily) to prevent infection during the study period.

### Image acquisition and processing

**Figure 1:**
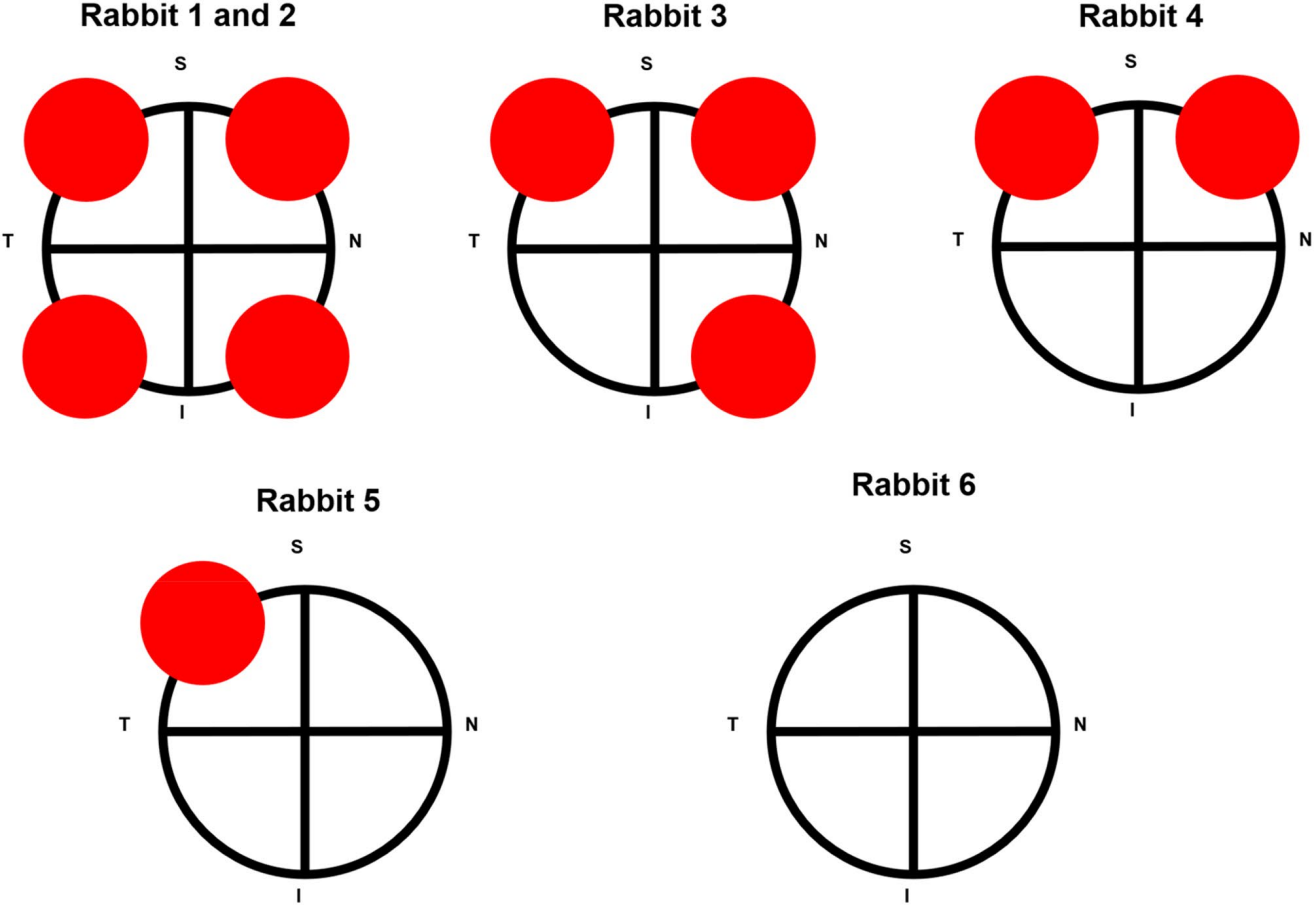
Schematic illustration of chemical ocular injury induced by alkali burns (represented by red circles as alkali-soaked filter paper discs, not to scale) using a previous described method^23^. N = Nasal. T = Temporal. S = Superior. I = Inferior.

**Figure 2:**
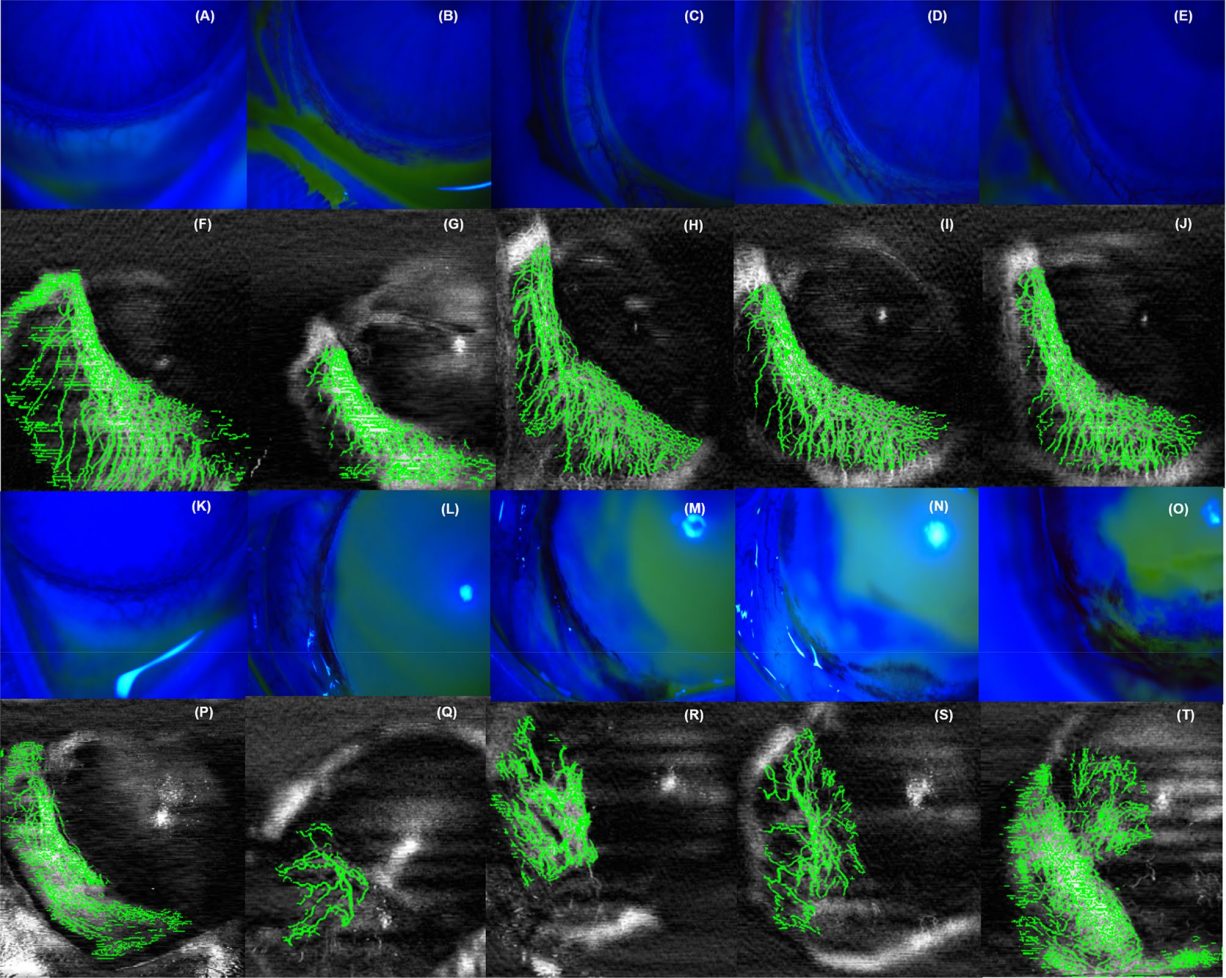
Representative images of serial anterior segment optical coherence tomography angiography and slit-lamp photography of control (**A**–**J**) and chemical injury (**K**–**T**) groups across various timepoints. **A**,**F**,**K**, and **P** were taken at baseline; **B**,**G**,**L**, and **Q** were taken Day-1 post-injury, **C**,**H**,**M**, and **R** were taken Day-3 post-injury. **D**,**I**,**N**, and **S** were taken Week-1 post-injury. **E**,**J**,**O** and **T** were taken Month-1 post-injury.

All rabbits were assessed under anaesthesia at baseline and the following time-points of post-injury: day 1, day 3, week 1 and 1 month. The corneas were analyzed via slit-lamp and AS-OCTA according to the four quadrants as illustrated in Fig. 1. Slit-lamp photography was obtained using a digital slit-lamp camera (Righton NS-2D, Tohoku Right Mfg, Japan) with a standard diffuse illumination (× 12 to × 35 magnification) (Fig. 2A–E; K–O). A scanning area of 9 mm × 9 mm (256 × 256 A-scans) of each region was taken with the Spectralis-domain (SD) OCT machine (central wavelength: 880 nm) with a lateral resolution of 20um, and axial resolution of 7um (Nidek Co Ltd., Gamagori, Aichi, Japan) (Fig. 2F–J; P–T). AS-OCTA images at all timepoints were exported and manually adjusted using the review software (Nidek Co Ltd., Gamagori, Aichi, Japan) to exclude the iris vessels from the enface OCTA images, leaving only the enface OCTA the projection slab of cornea for analysis. We further processed the images using a written program in MATLAB (Mathworks, Inc., Natick, MA, USA), similarly as described previously by our study group^20,21,25^. Briefly, we first removed the speckle noise using a median filter and Gaussian smoothing, then used Frangi filter to enhance vessel features^26^. Subsequently, local adaptive thresholding was used to segment vessels, and, a manually delineated mask was lastly applied to remove noise based on unconnected pixels. In the binarized images, white pixels represented the blood vessels, whilst black pixels represented the background; both were used to compute the vessel density (VD). VD was defined as the percentage of area of white pixels, out of the entire area of the binarized image, ie. the percentage area of limbal vessels within the region of interest—‘1’ for black pixels over the vessels, ‘0’ for the white background. It represented the overall quantitative value of perfused vessels in that en face image.

### Statistical analysis

IBM SPSS Statistics for Windows, version 26 (IBM Corp., Armonk, N.Y., USA) was used for statistical analysis in this study. One-way ANOVA model was used to compare the mean AS-OCTA VD across various timepoints with baseline VD within individual group. Independent t-test was used to compare the mean AS-OCTA between both groups (chemically-injured quadrants vs control quadrants in various eyes) at each given timepoint. Linear regression analysis was performed to analyze the relationship between AS-OCTA VD and the number of chemically-injured quadrants (out of four) at day 1 post-injury. The significance level was set at *p* < 0.05.

## Results

Overall, 48 quadrants of the eyes from the 12 eyes from the 6 rabbits underwent imaging, allowing us to obtain 240 slit-lamp photographs and 240 OCTA scans at various timepoints (baseline, day 1, day 3, week 1 and 1 month). Of the 48 quadrants, 28 quadrants were in the injury group and 20 were in the control group.

**Figure 3:**
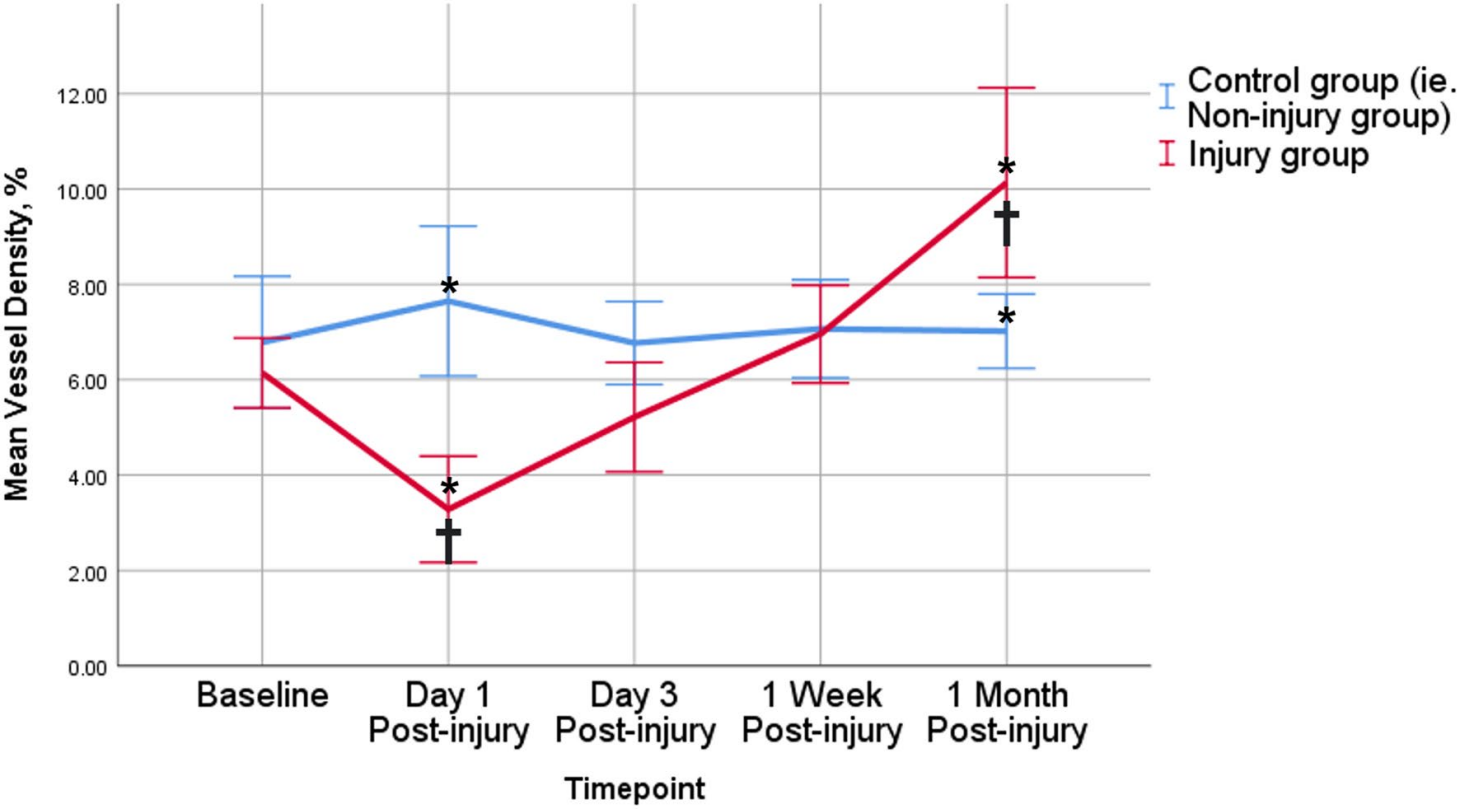
Comparison of changes in mean anterior segment-optical coherence tomography angiography vessel density (AS-OCTA) over time in both chemical injury and control quadrants. Error bar represents 95% confidence interval. *Statistically significant difference in mean vessel density between chemical injury and control groups at the same timepoint.

**Figure 4:**
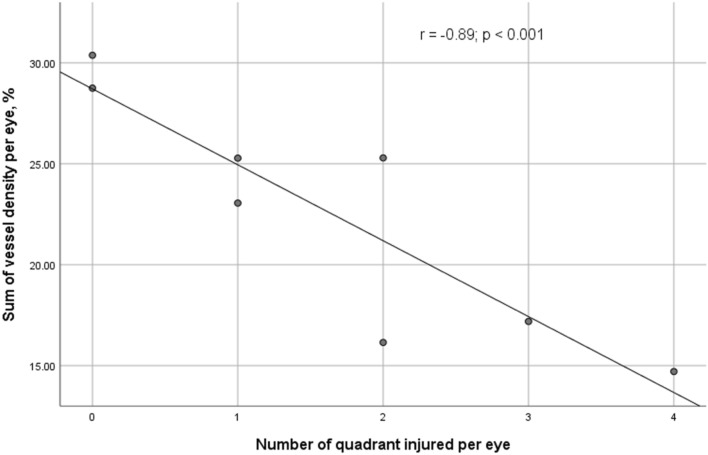
Scatter plot of anterior segment-optical coherence tomography angiography vessel density (AS-OCTA) values against the number of quadrants injured per eye.

At baseline, AS-OCTA VD was comparable between injury (6.1 ± 1.6%) and control groups (6.8 ± 2.1%; *p* = 0.34). AS-OCTA VD was significantly reduced in the injury group (3.3 ± 2.4%) compared to the control group (7.6 ± 2.3%), just after the insult at day 1 (*p* < 0.001). At 1 month post-injury, the VD of the injury group (10.1 ± 4.3%) became significantly higher than that of the control group (7.0 ± 1.2%; *p* = 0.025) (Fig. 3). AS-OCTA VD day 1 post-injury significantly correlated with number of quadrants with chemical injury (r = − 0.89; *p* < 0.001; 95% CI − 5.65 to − 1.87) (Fig. 4).

Significant changes in AS-OCTA VD within the chemical injury group was detected (*p* < 0.001). AS-OCTA VD of the injury group day 1 post-injury (3.3 ± 2.4%) injury group day 3 (5.2 ± 2.5%) and week 1 (7.0 ± 2.2%) post-injury were comparable with baseline (*p* = 0.99 and *p* = 0.99 respectively). AS-OCTA VD of injury group month 1 (10.1 ± 4.3%) post-injury was significantly higher when compared to baseline (6.1 ± 1.6%; *p* = 0.005). There was no significant difference across various timepoints for AS-OCTA VD of control group when compared to baseline (6.8 ± 2.1%) at day 1 (7.6 ± 2.3%), day 3 (6.8 ± 1.3%), week 1 (7.1 ± 1.5%), and 1 month (7.0 ± 1.2%) post-injury (*p* = 0.99 for all).

Specifically, when we compared AS-OCTA VD day 1 and month 1 post-injury, we found that amongst the injured group, AS-OCTA VD day 1 (3.3 ± 2.4%) was significantly lower when compared to 1 month (10.1 ± 4.3%; *p* < 0.001). In contrast, we did not find any significant difference within control quadrants (6.8 ± 2.1% vs 7.0 ± 1.2%; *p* = 0.99).

## Discussion

In this pilot animal study, we observed that AS-OCTA was able to detect a reduction in limbal VD within 24 h after acute chemical injury, and that the VD reduction significantly correlated with the extent of limbal alkali exposure. The AS-OCTA was also able to detect the development of corneal vascularization 1 month after the chemical injury. Our findings suggest that AS-OCTA may be a useful non-invasive imaging tool in acute chemical injury as it objectively detects limbal vessel disruption in such a setting and subsequent corneal vascularization at 1 month post-injury. As this is a pilot animal study, our aim was to first establish the role of AS-OCTA in detection of limbal vessel disruption. The advantage of this animal study lies in its ability to control the exact extent and location of alkali exposure to the limbus in each eye to correlate with AS-OCTA findings, which would be extremely difficult to do in a prospective clinical study especially in the emergency setting.

Limbal vessel disruption was detected by the AS-OCTA imaging in chemical-injured quadrants (or group) at day 1, while VD remained similar in the control quadrants (or group) when compared to baseline. This is consistent with a clinical study involving 15 human eyes (10 subjects) following acute chemical ocular injury, which demonstrated the use of AS-OCTA in detecting areas of limbal vessel disruption^22^. The study also suggested that AS-OCTA was able to reflect limbal disruption more extensively than clinical examination^22^, implying that clinical evaluation may underestimate the extent of limbal involvement post-injury. The AS-OCTA was also used to demonstrate the presence of limbal vessel disruption in a pilot clinical study^27^. However, both clinical studies were limited by a variable degree of limbal involvement and did not have control eyes to compare changes in limbal VD.

Our study using an animal model enabled us to use a gradated alkali exposure to specific quadrants of the cornea, allowing us to examine the use of AS-OCTA to assess specific grades of chemical ocular injuries. Our results suggested that AS-OCTA can quantify the extent of limbal disruption in various grades of chemical injuries. We noted that an increase in severity of chemical injury would lead to a corresponding fall in AS-OCTA VD at the acute phase. In the reparative phase of chemical injury, we also observed a significant increase in mean VD at 1 month post-injury compared to baseline. This likely reflects the development of secondary corneal vascularization due to the ensuing chronic inflammatory state from the release of cytokines such as vascular endothelial growth factors (VEGF) post-chemical injury^28–30^. Additionally, whilst dye angiography such as ICGA may be considered the current ‘gold standard’ for evaluation of vasculature in the anterior segment^31–33^, it is invasive and time-consuming which may not be easily implemented especially in the acute setting when managing chemical injuries. In contrast, AS-OCTA is a non-contact imaging technique that has been found comparable to ICGA, which can be used for serial adjunctive imaging even in the emergency clinical situation^20,21,34^.

The assessment of the corneal limbus in chemical ocular injury is an important surrogate to estimate the damage to the cornea limbal stem cells^35^. The two most commonly utilized classifications by Dua et al.^3^ and Roper-Hall^4^, incorporate clinical assessment of the extent of staining and limbal ischemia respectively post-injury using slit-lamp examination, which have been shown to be highly subjective^9^. As both classification systems have yet to be validated for use in animal models, we analyzed the strength of correlatability between the number of quadrants injured (instead of grade of injury) with the sum of mean VD from each quadrant of the corresponding eye. We observed a high level of correlation between the number of quadrants involved with AS-OCTA VD Day-1 post-injury (r = − 0.89; *p* < 0.001), suggesting that the AS-OCTA may be used to assess limbal involvement objectively. A previous study by our study group has suggested that AS-OCTA scans of chemical ocular injury has sustaintial agreement when assessing for limbal disruption as compared to slit-lamp evaluation^36^. Simiarly, Fung et al. demonstrated that AS-OCTA evaluation of limbal ischemia is usually more extensive than is suggested by clinical examination^22^. This showed that AS-OCTA may potentially be use to fill in the existing lacuna, ie. highly subjective assessment of chemical ocular injuries using slit-lamp by providing an objective lens. Hence, we have proposed a modified classification that allows for AS-OCTA assessment of limbal disruption (Table 1). This modified system aims to complement the current classification methods as in the current system, assessment of the conjunctiva under slit-lamp relies on an ordinal scale, eg. 9–< 12 clock hours of limbal involvement. However in reality, chemical ocular injuries may not follow such pattern. The incorporation of a continuous scale-based assessment may hence assists clinicians to assess chemical ocular injury and limbal involvement more accurately and objectively. This however requires a prospective clinical study to validate.

**Table 1:** Proposed classification of chemical ocular injury including anterior segment optical coherence tomography (AS-OCTA) detection of limbal involvement.

Grade	Slit-lamp evaluation	Conjunctival Fluorescein Stain	Limbal involvement detected by AS-OCTA
I	0 clock hours of limbal involvement	0%	0% ischemia
II	≤ 3 clock hours of limbal involvement	≤ 30%	> 0–< 25% ischemia
III	3–6 clock hours of limbal involvement	30 – 50%	25– < 50% ischemia
IV	6–9 clock hours of limbal involvement	50 – 75%	50– < 75% ischemia
V	9–< 12 clock hours of limbal involvement	75—< 100%	75–< 100% ischemia
VI	Total (12 clock hours) limbal involvement	100%	100% ischemia

This study has several limitations. Firstly, our study involved a small sample size of animal model. Despite that, through serial imaging, we obtained a sufficient number of images, and sufficient power to compare the AS-OCTA VD between groups. Secondly, we have only investigated the association of AS-OCTA VD with the extent of chemical injury involved per eye in our rabbit models, and recognize that the depth of limbal ischemia and corneal vascularization could be different with different types of OCTA devices used and animal species^37^. Lastly, we have primarily focused on only one AS-OCTA parameter, namely limbal vessel density. There exists other quantitative AS-OCTA vascular parameters as well^10^, e.g. vessel width, vessel branching, and validation of these parameters in OCTA assessment of limbal vessel disruption in chemical injury have yet to be explored. Further studies using larger sample sizes that could include other AS-OCTA parameters are required to validate the findings of this study.

In conclusion, using an established animal model of chemical ocular injury, our study suggests that AS-OCTA may be a useful and objective tool to assess limbal ischemia in the acute phase and detect corneal vascularization during the reparative phase. We propose that as a non-contact imaging tool, the AS-OCTA grading of limbal involvement may be incorporated into the acute care setting and improve objective assessment of acute chemical ocular injury. Previous clinical studies have suggested a role of AS-OCTA in the clinical assessment of acute chemical ocular injury, and our study supports the need for long-term prognostic studies to confirm its potential use in the proposed grading system.
